# Supplementary material for: Heterogeneous Transcriptional Landscapes in Human Sporadic Parathyroid Gland Tumors
Source: Int J Mol Sci. 2024 Oct 7;25(19):10782. doi: 10.3390/ijms251910782 (PMC11476768; doi:10.3390/ijms251910782)

Figure S1. a) Graphic representing interrelationships among the 7 genes selected for the unsupervised clustering analysis; red balls identified genes interacting in the RUNX3/WNT signaling; green balls identified genes interacting in the parathyroid-specific secretory pathway. b) Reported co-expression in Homo sapiens of the protein-coding genes investigated in the present study. Data are from STRING, version 12.0.

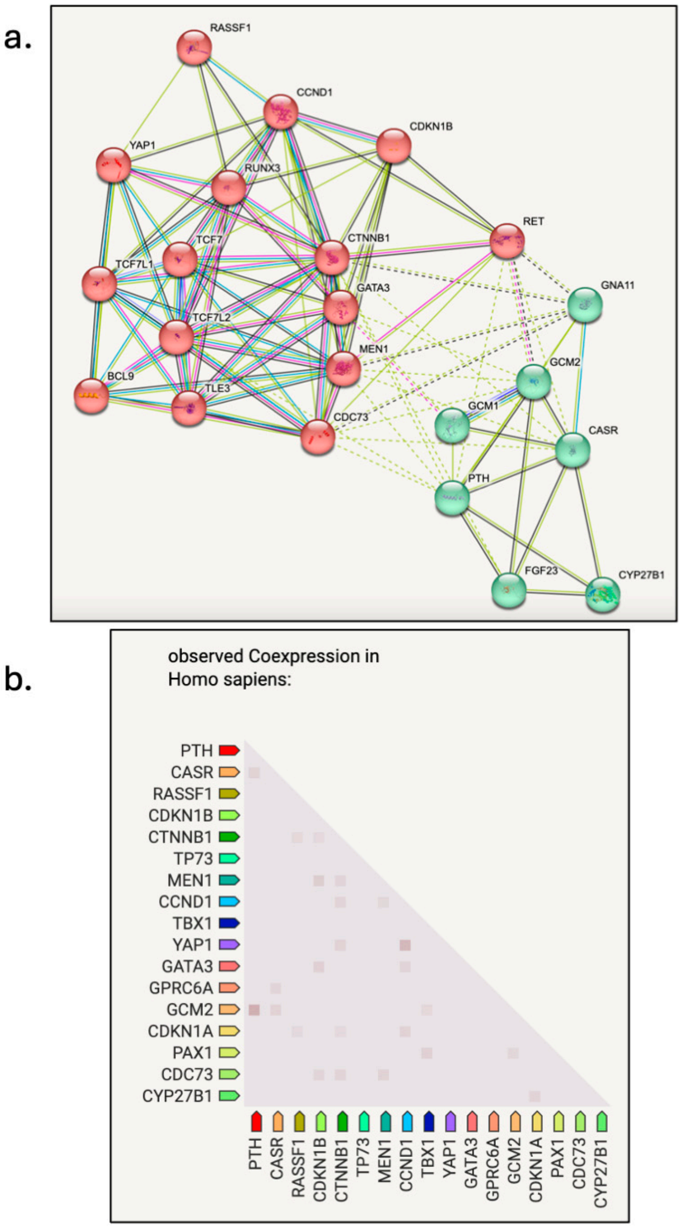

Figure S2. The connections identified in the C2B PAd pathways are involved in cell proliferation, the G0 to G1 phase transition, and adipogenesis (a,b).

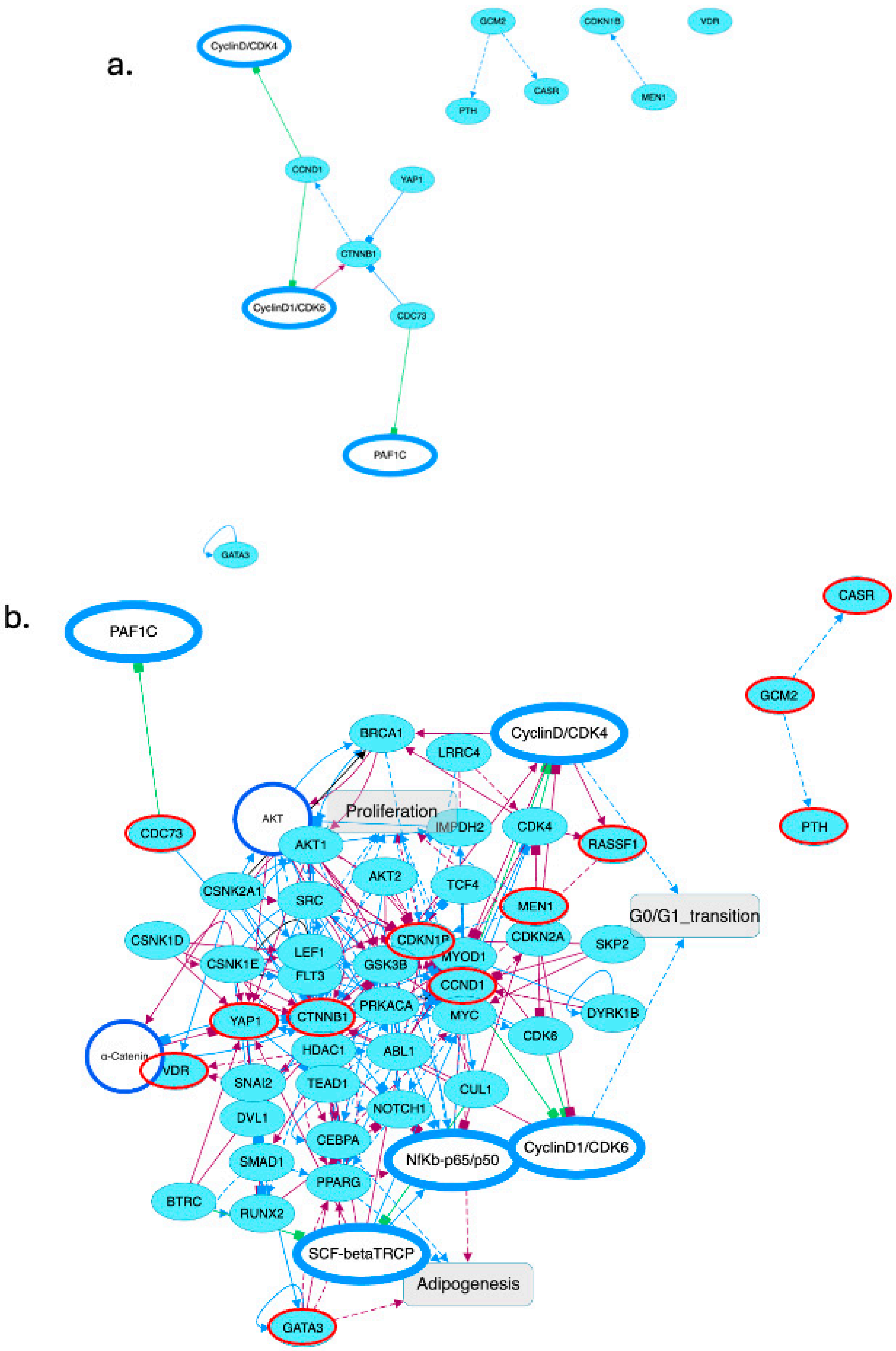

Figure S3. C2A PAds showed the deregulation of non-interconnected genes, though both were involved in the regulation of cell proliferation and cell cycle progress/block (a,b).

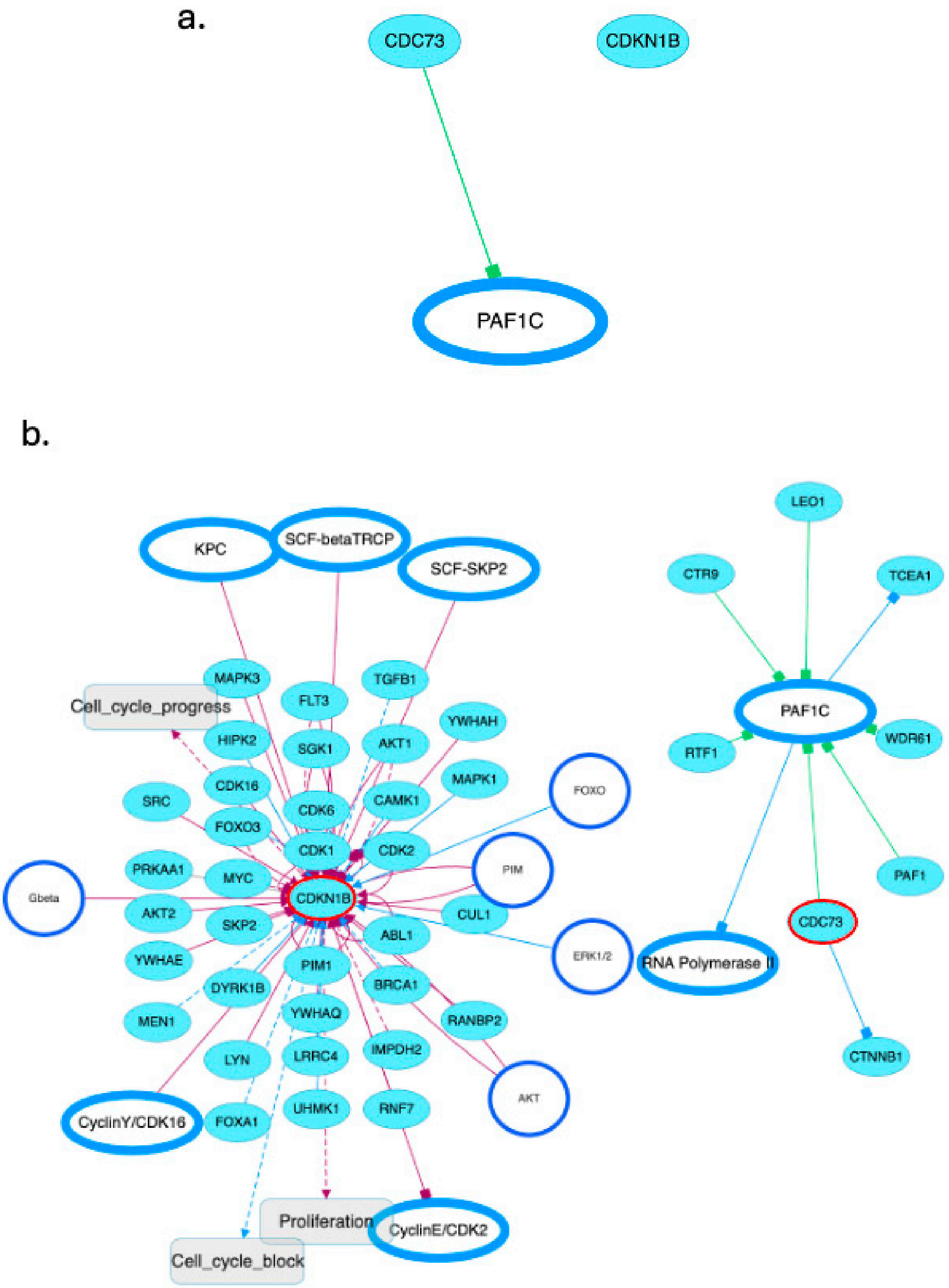

Supplement: Supplementary file 1 [file ijms-25-10782-s001.zip › Supplementary Figures S1-S3.pdf]
