# Supplementary material for: Heterogeneous Transcriptional Landscapes in Human Sporadic Parathyroid Gland Tumors
Source: Int J Mol Sci. 2024 Oct 7;25(19):10782. doi: 10.3390/ijms251910782 (PMC11476768; doi:10.3390/ijms251910782)
Supplement: Supplementary file 1 [file ijms-25-10782-s001.zip › Supplementary Table S1.pdf]

Supplementary Table S1.

Gene	Chromosome location	Protein	References
Oncosuppressors			
MEN1	**11**: 64,803,510-64,811,294	Menin	28
CDC73	**1**: 193,121,983-193,254,815	Parafibromin	3, 33, 34
RASSF1	**3**: 50,329,782-50,340,980	Ras association F1A	35, 36
YAP1	**11**: 102,110,447-102,233,424	Yes-associated	28
CTNNB1	**3**: 41,194,741-41,260,096	β-catenin	28
Embryonic transcription factors			
GCM2	**6**: 10,873,223-10,882,041	Glial-Cell Missing 2	4, 5, 6
TBX1	**22**: 19,756,703-19,783,593	T-box transcription factor 1	37
PAX1	**20**: 21,705,659-21,718,481	Paired box 1	38
GATA3	**10**: 8,045,378-8,075,198	GATA binding protein 3	39, 40
Parathyroid membrane receptors			
PTH	**11:** 13,492,054-13,496,181	Parathormone	41
CASR	**3**: 122,183,668-122,291,629	Calcium-sensing receptor	42, 43
VDR	**12**: 47,841,537-47,943,048	Vitamin D receptor	14
GPRC6A	**6**: 116,792,085-116,829,083	Osteocalcin receptor	44
Cell cycle regulatory genes			
CCND1	**11**: 69,641,156-69,654,474	Cyclin D1	45
CDKN1B	**12**: 12,685,498-12,722,369	p27	46
CDKN1A	**6**: 36,676,460-36,687,337	p21	47
TP73	**1**: 3,652,516-3,736,201	p73	44, 48
Long non-coding RNAs			
HAR1B	**20**: 63,090,806-63,102,631	-	49
HOXA-AS3	**7**: 27,129,977-27,155,928	-	49
HOXA-AS2	**7**: 27,107,777-27,134,302	-	49
NEAT1	**11**: 65,422,774-65,445,540	-	49
VLDLR-AS1	**9**: 2,421,597-2,622,457	-	49
SNHG6	**8**: 66,920,561-66,926,398	-	49
MicroRNAs			
MIR372	**19**: 53,787,890-53,787,956	-	32, 50
MIR517C	**19**: 53,741,313-53,741,407	-	32
MIR126	**9**: 136,670,602-136,670,686	-	51
MIR93	**7**: 100,093,768-100,093,847	-	52

Genes are annotated according to GRCh38:CM000682.2 by Ensembl
